# Relation between brain temperature and white matter damage in subacute carbon monoxide poisoning

**DOI:** 10.1038/srep36523

**Published:** 2016-11-07

**Authors:** Shunrou Fujiwara, Yoshichika Yoshioka, Tsuyoshi Matsuda, Hideaki Nishimoto, Akira Ogawa, Kuniaki Ogasawara, Takaaki Beppu

**Affiliations:** 1 Department of Neurosurgery, Iwate Medical University, 19-1 Uchimaru, Morioka, Iwate 020-8505, Japan; 2 Laboratory of Biofunctional Imaging, WPI Immunology Frontier Research Center, Osaka University, 3-1 Yamadaoka, Suita, Osaka 565-0871, Japan; 3 MR Applications and Workflow Asia Pacific, GE Healthcare Japan, 4-7-127 Asahigaoka, Hino, Tokyo 191-8503, Japan; 4 Department of Hyperbaric Medicine, Iwate Medical University, 19-1 Uchimaru, Morioka, Iwate 020-8505, Japan

## Abstract

In the previous studies, carbon monoxide (CO) poisoning showed an imbalance between cerebral perfusion and metabolism in the acute phase and the brain temperature (BT) in these patients remained abnormally high from the acute to the subacute phase. As observed in chronic ischemic patients, BT can continuously remain high depending on impairments of cerebral blood flow and metabolism; this is because heat removal and production system in the brain may mainly be maintained by the balance of these two factors; thus, cerebral white matter damage (WMD) affecting normal metabolism may affect the BT in patients with CO poisoning. Here, we investigated whether the BT correlates with the degree of WMD in patients with subacute CO-poisoning. In 16 patients with subacute CO-poisoning, the BT and degree of WMD were quantitatively measured by using magnetic resonance spectroscopy and the fractional anisotropy (FA) value from diffusion tensor imaging dataset. Consequently, the BT significantly correlated with the degree of WMD. In particular, BT observed in patients with delayed neuropsychiatric sequelae, a crucial symptom with sudden-onset in the chronic phase after CO exposure, might indicate cerebral hypo-metabolism and abnormal hemodynamics like “matched perfusion,” in which the reduced perfusion matches the reduced metabolism.

Brain temperature (BT) can be altered by the abnormal imbalance of two factors, the heat produced by cerebral metabolism and its removal by the cerebral blood flow (CBF)^1^, called the ‘heat removal’ theory. It has already been reported that BT elevation occurred in patients presenting abnormal cerebral hemodynamics such as acute stroke, chronic ischemia, and moyamoya disease^2^,^3^,^4^. Especially, patients who showed BT elevation commonly might have an accompanying condition called ‘misery perfusion’^5^,^6^. Misery perfusion involves a cerebral hemodynamic situation with reduced CBF and a maintained cerebral metabolic rate of oxygen (CMRO_2_), which indicates an elevated oxygen extraction fraction (OEF). Carbon monoxide (CO) poisoning also causes strong imbalance in cerebral hemodynamics like misery perfusion, and an OEF elevation has been demonstrated using positron emission tomography (PET) with ^15^O-gas^7^, which is a gold standard modality for estimating the state of cerebral perfusion and metabolism. BT elevation in patients with CO-poisoning has clearly been observed in the acute phase, and the abnormal BT elevation persists until the subacute phase^8^. On the other hand, patients who presented delayed neuropsychiatric sequelae (DNS) showed remarkable BT reduction of >1 °C between the acute and subacute phases. It has been well known that the occurrence of DNS was highly associated with cerebral white matter damage (WMD) caused by progressive demyelination after CO inhalation^9^,^10^,^11^,^12^,^13^; thus, BT in patients with CO-poisoning may potentially be associated with the degree of WMD, which can affect brain metabolism. Thus, the aim of the present study was to investigate whether the BT in patients with CO-poisoning was associated with the degree of WMD in the subacute phase.

## Results

**Figure 1:**
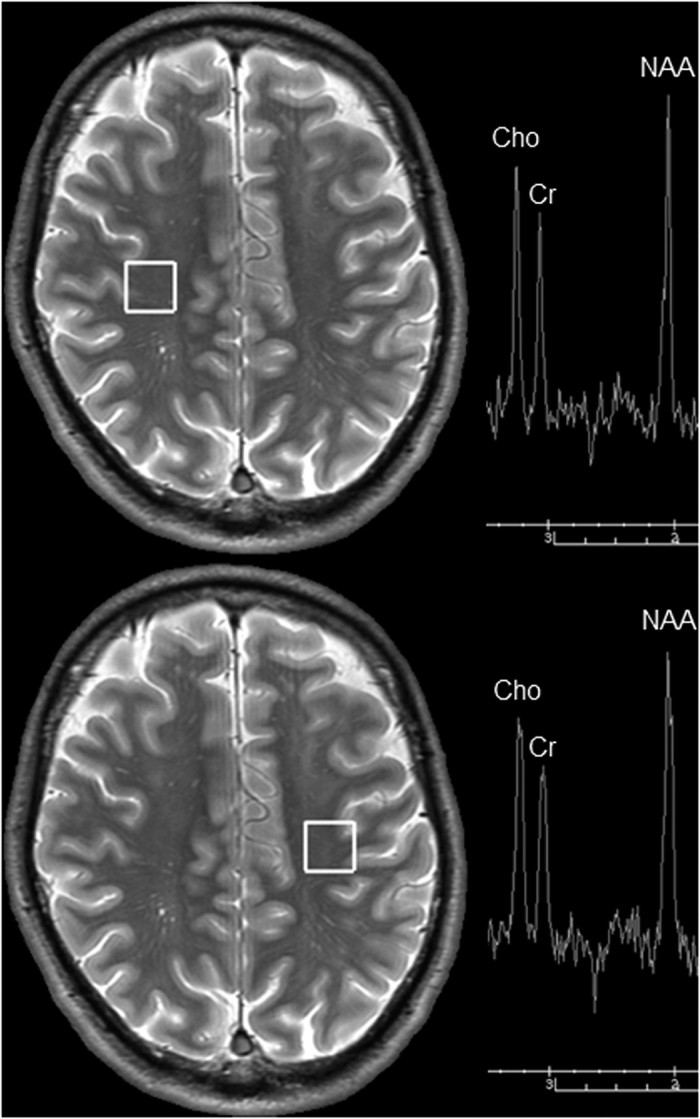
Region of interest location and typical spectra for magnetic resonance spectroscopy measurement in a patient showing delayed neuropsychiatric sequelae on day 27 from admission (case 16). Cho, choline; Cr, creatine; NAA, *N*-acetylaspartate.

**Figure 2:**
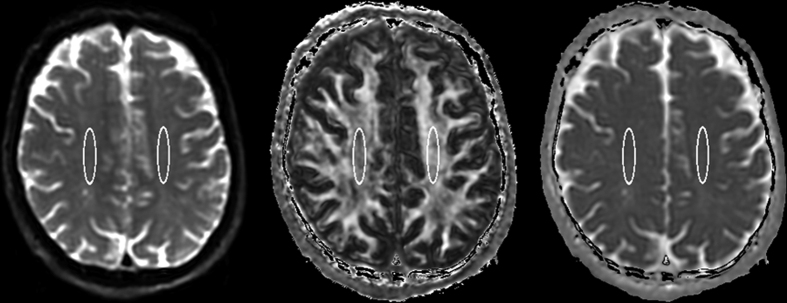
Region of interest location defined on the non-diffusion weighted image (b = 0 mm^2^/s) for the measurement of fractional anisotropy (middle) and apparent diffusion coefficient (right) in a patient showing delayed neuropsychiatric sequelae on day 27 from admission (case 16).

The clinical data and each parameter for all 16 patients are summarized in Table 1. All patients completely recovered from the initial symptoms within 3 days after admission. Of these, thirteen patients (81%) showed no neuropsychiatric symptoms for 6 weeks. The remaining three patients (19%) showed DNS after lucid interval (LI) (case 8: 30 days, case 15: 26 days, and case 16: 27 days). All patients with DNS mainly showed cognitive dysfunction that continued until the 6th week (the mini-mental state examination scores at 6 weeks, case 8: 10, case 15: 22, and case 16: impracticable). We successfully performed magnetic resonance imaging (MRI) scans with good quality in all 16 patients; however, the body temperature on the day of MRI scan could be measured at the ward in only 13 patients because three patients (case 5, case 7, case 10) were discharged from the hospital before the day of MRI scan (Table 1). Figure 1 shows the typical volume of interest (VOI) locations and spectra of magnetic resonance spectroscopy (MRS) at the centrum semiovale on both sides, and Fig. 2 shows the region of interest (ROI) locations for the measurements of fractional anisotropy (FA) and apparent diffusion coefficient (ADC) from the diffusion tensor imaging (DTI) dataset (case 16). The intraclass correlation coefficient (ICC) for FA and ADC measurements indicated good agreement (0.8182 and 0.8938, respectively).

**Figure 3:**
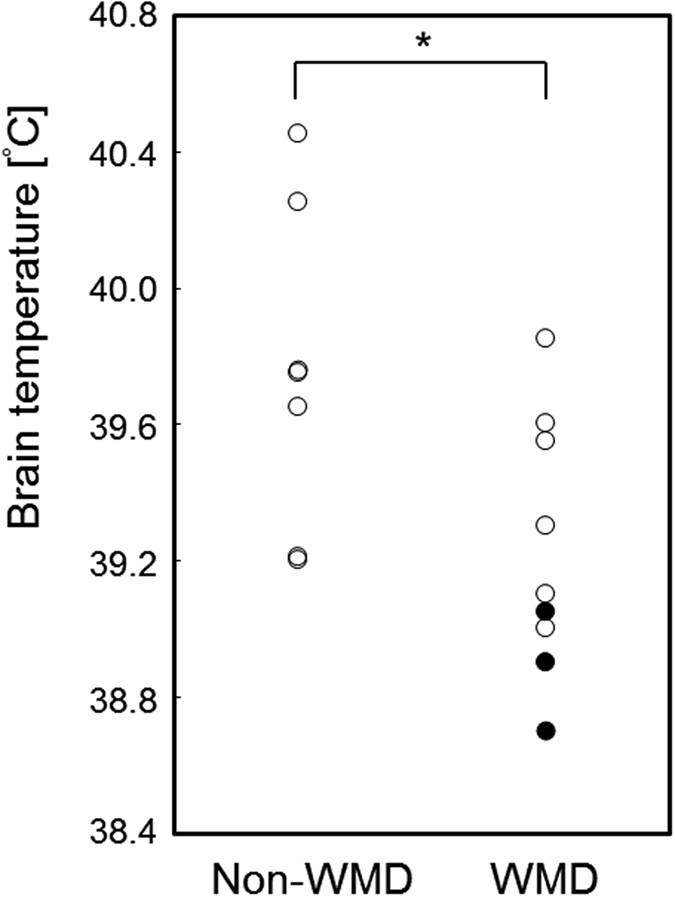
Brain temperature (BT) in carbon monoxide (CO)-poisoned patients at the subacute phase. The BT in subacute CO-poisoned patients was higher than the cut-off value of BT (38.3 °C) determined from a control group. The BT in the white matter damage (WMD) group, composed of patients who showed a fractional anisotropy value less than the cut-off value (0.374), was significantly lower than that in the non-WMD group. Black circles indicate patients who showed delayed neuropsychiatric sequelae (DNS) after a lucid interval; white circles indicate patients who showed no DNS but only initial symptoms within 3 days after admission.

**Figure 4:**
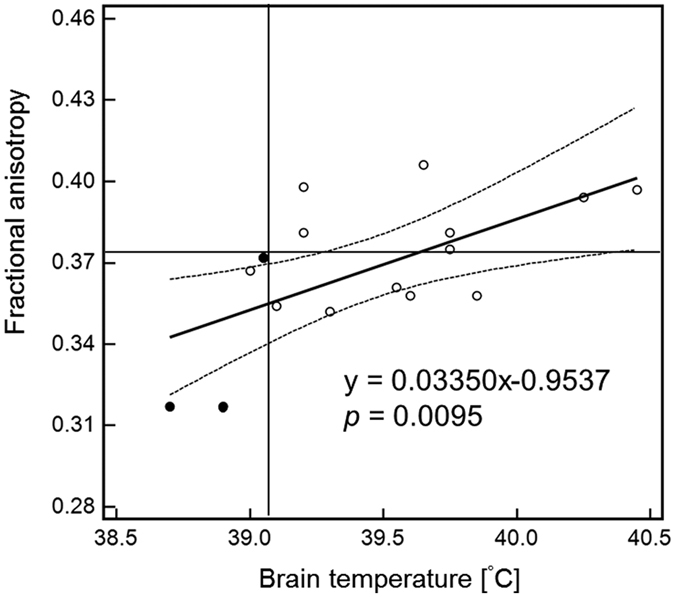
Relation between the brain temperature (BT) and fractional anisotropy (FA) value in all 16 CO-poisoned patients at the subacute phase. The BT significantly correlated (ρ = 0.542, *p* = 0.0302) and was associated (slope: 0.03350, *p* = 0.0480; intercept: −0.9537, *p* = 0.0095; *F*-test: *p* = 0.0095) with the FA value, and the dot curved lines indicate the 95% confidential interval. The horizontal and vertical lines indicate the cut-off values of FA (0.374) and BT (39.05 °C), respectively.

The BT was abnormally higher in all 16 patients with CO-poisoning, and 9 and 7 patients (56% and 44%, respectively) were assigned to the WMD group (7 men and 2 women; mean age: 41.6 ± 14.5 years; age range: 23–71 years) and the non-WMD group (7 men; mean age: 51.7 ± 18.6 years; age range: 30–72 years), respectively, with a cut-off FA value (0.374) from healthy subjects (Table 1). All three patients with DNS were included in the WMD group. The BT in the non-WMD group was significantly higher than that in the WMD group (*p* = 0.0311; Fig. 3). When we defined 39.05 °C as the cut-off value by receiver operating characteristic (ROC) curve analysis, BT was a good predictor for distinguishing all patients with DNS from other patients (sensitivity: 100%; specificity: 92.3%; positive predictive value: 75.0%; negative predictive value 100%, area under curve: 0.974; confidential interval: 0.752–1.00; *p* < 0.0001; Fig. 4). Additionally, BT was significantly correlated with the FA value (ρ = 0.542, *p* = 0.0302), and linear regression analysis indicated a significant association (slope: 0.03350, *p* = 0.0480; intercept: −0.9537, *p* = 0.0095; *F*-test: *p* = 0.0095; Fig. 4); however, there was no significant correlation between BT and ADC (ρ = −0.279, *p* = 0.2953). Moreover, no significant correlations were found between age and BT or FA (ρ = 0.195, *p* = 0.4703; ρ = 0.192, *p* = 0.4769, respectively).

Body temperature, which could be measured in only 13 patients (Table 1), was significantly lower than BT (*p* < 0.0002) and no significant correlation was observed between the two parameters (ρ = 0.212, *p* = 0.4868). In addition, no significant difference in body temperature was observed between the WMD and non-WMD groups (*p* = 0.4633). Furthermore, there was no significant correlation between body temperature and FA (ρ = −0.249, *p* = 0.4114). On the other hand, ΔT ( = BT – body temperature) strongly correlated with FA (ρ = 0.733, *p* < 0.0043).

## Discussion

The main findings of this study are as follows: (1) increased BT correlates with the degree of WMD; (2) ΔT, which can represent pure BT effect by decreasing the effect of body temperature, more strongly correlates with the degree of WMD in subacute CO poisoning. In addition, the BT in patients with subacute CO-poisoning might be able to partially represent their brain metabolism because the BT was lower when WMD was stronger, as seen in patients with severe WMD showing DNS in the present study. We also confirmed that patients with subacute CO-poisoning displayed high BT compared to body temperature and that the BT was abnormally high compared to the normal BT from healthy controls as reported in a previous study^8^. Those findings suggested that the basic regulation system for BT might have a different mechanism from that for body temperature regulation, although the absolute BT value included a small effect of body temperature. No previous studies have examined this relationship in patients with CO-poisoning by estimating the BT by means of ^1^H-MRS and determining the FA value from the DTI dataset.

Brain temperature has traditionally been viewed as a parameter that passively reflects a collective state of brain activity. On the other hand, it has been reported that BT might also act as a control parameter with a dynamic fluctuation modulating brain activity and function^14^,^15^. In a recent animal study, it has been reported that BT in songbirds could easily be increased by singing faster tempo songs^16^, which suggested that subtle changes in neural activity might affect BT. Also in a human study, such sensitive response of BT to neural activity was observed with chemical shift-based BT measurement and functional MRI with visual stimulation^1^. The study also demonstrated that an increase in regional CBF during functional stimulation could cause a BT reduction by 0.2 °C and subsequent local changes in oxygen metabolism. The findings in humans indicated that CBF could be significantly associated with BT changes. However, when CBF continuously diminishes, BT would largely be defined by metabolic activity throughout the brain. In a previous study, CBF continuously decreased from 3 days to 1 month after CO exposure not only in patients showing DNS but also in those showing no neuropsychiatric sequelae^17^. In other words, CBF remained abnormally low until the subacute phase, even if the brain damage was mild. High OEF defined as misery perfusion, which indicates CBF reduction and maintained CMRO_2_, was also identified by means of PET in the acute phase after CO exposure^7^. Various reports from patient studies suggested that high BT could be associated with abnormal cerebral perfusion and metabolism, such as misery perfusion^1^,^2^,^3^,^4^. In fact, as seen in a previous report^8^, an abnormally high BT was observed in all patients with subacute CO-poisoning. Based on the heat removal mechanism in which heat produced by cerebral metabolism can be removed by CBF, high BT can be observed when CBF is reduced. Thus, high BT in the present study might suggest that the heat production by cerebral metabolism might exceed the heat removal by CBF. On the other hand, the mechanism for continuous CBF reduction has been unclear. However, the cerebrovascular autoregulation can be damaged by potent oxidative stress after CO exposure to the endothelial cells in the vascular lining during the acute phase^13^,^18^. Hence, CBF might be reduced, and then BT might depend strongly on cerebral metabolism, including the metabolism of glucose, oxygen, and active membrane at the subacute phase after CO exposure. Consequently, BT would remain high even in the subacute phase.

In contrast, the direct mechanism causing BT reduction from the acute and subacute phase observed in a previous study^8^ has been unclear. However, it is well known that the subacute phase is an important phase that indicates a risk of developing DNS in patients after CO exposure, and that WMD is highly associated with the occurrence of DNS^9^,^10^,^12^,^19^. Therefore, we hypothesized that BT is associated with WMD in patients with CO-poisoning. Consequently, two patients with DNS with severe WMD, according to low FA value, showed low BT compared to most other patients in the present study, although their BTs were still higher than those of controls, even at the subacute phase. However, severe WMD could potentially decrease the BT to lower than the limit of the normal BT (38.3 °C) (called ‘pseudo-normalization’) because these patients showed severe neuropsychiatric symptoms, that is, DNS, in the chronic phase. Thus, these patients might have an abnormal state due to reduced metabolism matching reduced CBF, like matched perfusion in patients with chronic ischemia^20^. If this is true, then the BT reduction during the period between the acute and subacute phases in a previous study^8^ might be mainly due to WMD, that is, a metabolic reduction.

In the present work, no dynamic BT fluctuation measurement in real-time was performed; however, we might incidentally indicate that BT was associated with brain function by assessing BT in DNS patients. In general, DNS patients show severe cognitive impairment at the chronic phase and the impairment may be mainly caused by progressive demyelination^10^,^13^, which may start at the earlier phase. Low FA value of DNS patients observed in the present work indicates cerebral white matter damage caused by such progressive demyelination. Consequently, the cerebral metabolism associated with bran function might be reduced in DNS patients by the cerebral white matter damage. Hypometabolism might cause low BT; thus, low BT might reflect cerebral hypometabolism causing the impairment of brain function as seen in DNS patients.

The present study could demonstrate that BT was significantly higher than body temperature, as seen in Table 1, and no significant correlation was observed between the two temperatures in subacute CO poisoning. It suggested that BT might have a different energy source and removal system from that of body temperature; that is, different regulation mechanisms might exist for BT and body temperature. As seen in the present and previous works, a remarkably abnormal state like CO poisoning or ischemia might sharply mark the basic difference in the mechanism between BT and body temperature. Although there were few reports about direct BT measurement in human, the difference between the two temperatures has already been reported^21^. In a previous work, BT measurement was performed by monitoring the ventricular catheter of patients with subarachnoid hemorrhage (SAH) and they were assigned into different groups based on the difference between BT and body temperature (like ΔT in the present study). The report demonstrated that the higher BT group showed good outcome after SAH and all patients in the lower BT group died. In these results, the lower BT might reflect a remarkably low metabolism in the brain and the main regulation system for BT might be independent of that of body temperature. Furthermore, another previous study has reported a good significant correlation between BT and CMRO_2_ in patients with chronic ischemia^3^. Considering the large discrepancy between the abnormal high BT and normal body temperature in previous studies, it is natural to think that the basic mechanism to regulate BT might be different from that for body temperature, at least when the brain is in an abnormal state like low perfusion, low metabolism, and/or hypoxia after strong damage like SAH, ischemia, and CO poisoning^3^,^8^,^21^,^22^,^23^.

It may be difficult to clearly distinguish the main mechanisms underlying the two temperatures because of the complex relationship, where small differences are difficult to estimate, especially in healthy volunteers^24^. Even during normal neuronal activation in the brain, if CBF can be increased according to oxygen consumption in the activated areas^1^, BT could regionally be decreased because of increased heat removal exceeding increased heat production. In a broad perspective, it has already been reported that the mean BT and tympanic temperature are independent of each other in humans^25^. Indeed, this relationship also is now unclear; however, a special state such as that showing OEF elevation and/or severe WMD might clarify the different mechanisms because the resulting abnormal situation could be a key to separately estimate the difference between body temperature and BT. Because of such an abnormal state, conventional relationships between age and BT or FA might be hidden because aging can reduce the brain metabolism and cause mild demyelination in the white matter^26^,^27^.

The present study has some limitations. First, we used single-voxel MRS for BT measurement. Multi-voxel MRS has been suggested to be a more powerful tool for the visualization of temperature distribution than the single-voxel technique. Second, patients who showed persistent severe symptoms could not be enrolled because they were not admitted to the hospital. Third, the effect of inflammation on BT could not be clarified by using inflammatory markers such as interleukin-6^24^. Fourth, the actual situation of cerebral perfusion and metabolism could not be assessed by using PET, which is the gold standard for estimating cerebral hemodynamics. These need to be validated in further studies by means of quantitative comparisons between the BT and PET findings. Fifth, the relation between BT and brain activity in real-time has been unclear because no BT fluctuation measurement was performed in the present study.

In conclusion, the present study demonstrated that the BT was abnormally high in patients with CO-poisoning at the subacute phase, which was significantly correlated with the degree of WMD. The near-normal BT observed in patients with severe WMD, especially in patients with DNS, might indicate cerebral hypometabolism as seen in patients with “matched perfusion,” where the reduced perfusion matches the reduced metabolism.

## Methods

### Patients

Patients admitted to our institution between October 2009 and April 2011 were included in this study. The entry criteria for this study were as follows: provision of written informed consent to participate, from the patient or the patient’s family members; age <80 years and >20 years; diagnosis of CO poisoning caused by a fire or charcoal burning; no history of brain disorders, including surgical operation, irradiation, stroke, infection, remarkable atrophy, or demyelinating disease. The diagnosis was based on a current CO exposure and the presence of initial neurological symptoms on admission. Among the 25 patients admitted to our hospital suspected to have CO poisoning, a total of 16 patients (14 men, 2 women; mean age, 46.0 ± 16.6 years; age range, 23–72 years) who matched to the above criteria were enrolled. Nine patients were excluded owing to having another systemic disorder (connective tissue disease, n = 1), severe brain atrophy (n = 1), being older than >80 years (n = 3), having no good spectra (n = 1), and having no MRI scan at the subacute phase (n = 3). In each patient, we evaluated the initial neurological symptoms including level of consciousness by using the Glasgow coma scale and the percentage of carboxyhaemoglobin in arterial blood immediately after admission. All patients were treated with hyperbaric oxygen therapy started within 24 h of admission. The day of CO inhalation was defined as day 1 in this study. The neuropsychiatric symptoms were continuously observed during 6 weeks. DNS was defined by using the following two criteria: first, the onset was from 1 week to 6 weeks after LI, which is the period during which a DNS patient temporarily shows no neurological deficits and no cognitive impairments after recovering from the initial symptoms within 3 days; second, various characteristic symptoms of DNS appeared, such as Parkinsonism, urinary incontinence, dementia, and obvious personality change that the family can recognize. If DNS appeared in a patient, MMSE was performed for simply assessing general intellectual function^28^. Furthermore, evidences of obvious personality change according to interviews with the patient’s family were also investigated together with the diagnosis of dementia. All MRI procedures were performed for all 16 patients in the subacute phase (mean, 15.4 ± 2.0 days; range, 12–19 days), by using a 3-T MRI scanner (Signa Excite HD; GE Healthcare, Milwaukee, WI, USA) with an eight-channel head coil. Body temperature was measured at the ward before the MRI scan in each patient who stayed in the hospital until the day of MRI. All study protocols were approved by the Ethics Committee of Iwate Medical University, Morioka, Japan and were carried out in accordance with the approved relevant guidelines and regulations.

### BT measurement

Magnetic resonance spectra from single-voxel MRS were acquired by using point-resolved spectroscopy with the following parameters: repetition time (TR), 2000 ms; echo time (TE), 144 ms; and VOI, 1.5 × 1.5 × 1.5 cm^3^. Avoiding contamination of the signal by cerebrospinal fluid in the lateral ventricle and sulci, the VOI was carefully placed on the bilateral centrum semiovale in the deep cerebral white matter on an axial fast spin echo short-inversion time inversion recovery (FSE-STIR) image scanned before the MRS acquisition (TR/TE, 5600/23.2 ms; inversion time, 100 ms; matrix size, 512 × 256; field of view [FOV], 24 × 24 cm^2^; slice thickness, 4.0 mm with 1.5 mm interslice gaps; number of slices, 24; number of average, 1; parallel imaging reduction factor, 2). Raw data from MRS (4096 zero filling, 1 Hz apodization, and fast Fourier transform) were automatically analysed by using an automatic curve-fitting procedure and decomposed into Lorentzian peak components on the MR console. The BT for each VOI was calculated from the amount of the chemical shift difference between the water (H_2_O) and *N*-acetylaspartate (NAA) signals as Δ(H_2_O-NAA), by using the calibration data from Cady *et al.*, as follows: T [°C] = 286.9−94*Δ(H_2_O-NAA)^29^. The BT was calculated from the Δ(H_2_O-NAA) of MRS by using a custom-made temperature analysis software. Two BTs from the bilateral centrum semiovale in each patient were averaged, and the averaged value was defined as the BT in each patient. When a BT was higher than the cut-off value (38.3 °C), which was defined by the mean + 2 × standard deviations of the BTs from a control group (mean, 37.8 ± 0.28 °C; range, 37.3–38.2 °C) composed of 15 healthy subjects showing no pathological lesions (12 men, 3 women; mean age, 30.2 ± 6.2 years; age range, 21–50 years), the BT was defined as abnormally high.

### DTI for assessing the degree of WMD

After the MRS acquisition for a BT measurement, DTI was performed in all patients with the FOV, slice thickness, slice number, and slice position identical to those used for obtaining the FSE-STIR image. The other imaging parameters were as follows: axial single-shot spin-echo echo-planar imaging; TR/TE, 10,000/66 ms; motion-probing gradient directions, 6; b value, 0 and 1000 [s/mm^2^]; number of average, 3; parallel imaging reduction factor, 2. The FA and ADC maps were calculated in each patient from the DTI dataset after the correction of image distortion on the MRI console. The maps were transferred to a workstation and transformed from DICOM image format into ANALYZE image format by using free software (MRIcro, http://www.cabiatl.com/mricro/)^30^. Each FA and ADC value was measured at a slice including the centrum semiovale by using a same region of interest (ROI), which was manually placed by a single operator on the non-diffusion weighted (b = 0 s/mm^2^) image obtained with DTI acquisition. The ROI was automatically located on the FA and ADC map by using MRIcro. Each FA and ADC value at the centrum semiovale was measured twice to assess the intraclass correlation coefficient (ICC), which can validate the reliability of the measurement in an operator. The mean of two values, measured on the left and right sides, was defined as each FA or ADC value in each patient. All patients were assigned to one of two study groups according to the normal cut-off value for FA from a control group. The cut-off value was defined by the mean − standard deviations of the FA values (mean, 0.40 ± 0.03; range, 0.36–0.45) from 17 healthy subjects showing no pathological lesions (14 men, 3 women; mean age, 39.1 ± 10.6 years; age range, 22–58 years). A patient showing an FA value lower than the cut-off value (0.374) was assigned to the WMD group (including mild and severe WMD), whereas a patient showing an FA value higher than the cut-off value was assigned to the non-WMD group.

### Statistical analysis

The BT was compared between the WMD and non-WMD groups by using Mann-Whitney *U* test. To assess the relation between the BT and the FA value, ADC value, or body temperature, Spearman’s rank correlation coefficient was calculated. Then, linear regression analysis was performed to clarify the association between BT and other parameters, of which the rank correlation coefficient indicated the relation with the statistical significance. In addition, ROC curve analysis was performed to define an optimized BT at the subacute phase, to distinguish DNS patients from those showing no DNS. To confirm the effect of age on the BT and FA, Spearman’s rank correlation coefficient was calculated between age and BT or FA. Furthermore, we examined the difference between BT and body temperature with Wilcoxon signed-rank test. Next, we calculated ΔT ( = BT-body temperature) to exclude the body temperature effect on BT, and examined the correlation between ΔT and FA with Spearman’s rank correlation coefficient. All statistical procedures were performed with MedCalc statistical software version 13.1.2 (MedCalc Software bvba, Ostend, Belgium; http://www.medcalc.org; 2014), and the significance was examined at the *p* < 0.05 level.

## Additional Information

**How to cite this article**: Fujiwara, S. *et al.* Relation between brain temperature and white matter damage in subacute carbon monoxide poisoning. *Sci. Rep.* **6**, 36523; doi: 10.1038/srep36523 (2016).

**Publisher’s note:** Springer Nature remains neutral with regard to jurisdictional claims in published maps and institutional affiliations.

## Figures and Tables

**Table 1:** Characteristics and clinical data of CO-poisoned patients.

Group	No	Age (years)	M/F	COHb (%)	GCS	Symptom	Day of MRI	FA	Body T (°C)	BT† (°C)	ΔT (°C)
Non-WMD	1	34	M	38.6	5	IC	16	0.406	36.8	39.65	2.85
	2	65	M	16.3	12	IC	18	0.398	36.1	39.20	3.10
	3	56	M	12.2	15	Headache	15	0.397	36.7	40.45	3.75
	4	34	M	44.1	10	IC	15	0.394	36.5	40.25	3.75
	5	72	M	29.4	13	IC	15	0.381	—	39.75	—
	6	30	M	37.9	14	IC	18	0.381	36.7	39.20	2.50
	7	71	M	25	11	IC	15	0.375	—	39.75	—
WMD*	8‡	23	F	37.1	10	IC	12	0.372	36.6	39.05	2.45
	9	71	M	53	4	IC	15	0.367	36.3	39.00	2.70
	10	51	F	35.5	10	IC	19	0.361	—	39.55	—
	11	36	M	23.5	10	Dizziness	13	0.358	37.0	39.60	2.60
	12	32	M	19.3	10	Headache	18	0.358	36.7	39.85	3.15
	13	31	M	47.3	13	IC	15	0.354	36.5	39.10	2.60
	14	48	M	44.0	6	IC	14	0.352	37.0	39.30	2.30
	15‡	34	M	3.4	8	IC	14	0.317	37.3	38.90	1.60
	16‡	48	M	28.6	6	IC	14	0.317	36.5	38.70	2.20

WMD, white matter damage; COHb, carboxyhaemoglobin; GCS, Glasgow coma scale; MRI, magnetic resonance imaging;

FA, fractional anisotropy; Body T, body temperature; BT, brain temperature; IC, impairment of consciousness.

–: no examination or no data.

^*^The FA value of patients in the WMD group was lower than the mean + standard deviation (0.374) of FA values from the healthy subjects.

^†^The BT of all patients was higher than the mean + 2 standard deviation (38.3 °C) of the BT determined from the healthy subjects.

^‡^Patients showing delayed neuropsychiatric sequelae.
